# Upregulation of TH/IL-17 Pathway-Related Genes in Human Coronary Endothelial Cells Stimulated with Serum of Patients with Acute Coronary Syndromes

**DOI:** 10.3389/fcvm.2017.00001

**Published:** 2017-02-07

**Authors:** Giovanni Cimmino, Loreta Pia Ciuffreda, Giovanni Ciccarelli, Paolo Calabrò, Fiorella Angelica Valeria Ferraiolo, Alessia Rivellino, Raffaele De Palma, Paolo Golino, Francesco Rossi, Plinio Cirillo, Liberato Berrino

**Affiliations:** ^1^Department of Cardio-Thoracic and Respiratory Sciences, Section of Cardiology, University of Campania “Luigi Vanvitelli”, Naples, Italy; ^2^Department of Experimental Medicine, Section of Pharmacology, University of Campania “Luigi Vanvitelli”, Naples, Italy; ^3^Department of Clinical and Experimental Medicine, Section of Immunology, University of Campania “Luigi Vanvitelli”, Naples, Italy; ^4^Department of Advanced Biomedical Sciences, Section of Cardiology, University of Naples, “Federico II”, Naples, Italy

**Keywords:** acute coronary syndrome, atherosclerosis, endothelial cells, gene expression, immunity, Th-17

## Abstract

**Background:**

Inflammation plays an essential role in the development and complications of atherosclerosis plaques, including acute coronary syndromes (ACS). Indeed, previous reports have shown that within the coronary circulation of ACS patients, several soluble mediators are released. Moreover, it has been demonstrated that endothelial dysfunction might play an important role in atherosclerosis as well as ACS pathophysiology. However, the mechanisms by which these soluble mediators might affect endothelial functions are still largely unknown. We have evaluated whether soluble mediators contained in serum from coronary circulation of ACS patients might promote changes of gene profile in human coronary endothelial cells (HCAECs).

**Methods:**

HCAECs were stimulated *in vitro* for 12 h with serum obtained from the coronary sinus (CS) and the aorta (Ao) of ACS patients; stable angina (SA) patients served as controls. Gene expression profiles of stimulated cells were evaluated by microarray and real-time PCR.

**Results:**

HCAECs stimulated with serum from CS of ACS patients showed a significant change (upregulation and downregulation) in gene expression profile as compared with cells stimulated with serum from CS of SA patients. Moreover, *ad hoc* sub analysis indicated the upregulation of Th-17/IL-17 pathway-related genes.

**Conclusion:**

This study demonstrates that, in ACS patients, the chemical mediators released in the coronary circulation might be able to perturb coronary endothelial cells (ECs) modifying their gene profile. These modified ECs, through downregulation of protective gene and, mainly, through upregulation of gene able to modulate the Th-17/IL-17 pathway, might play a key role in progression of coronary atherosclerosis and in developing future acute events.

## Introduction

Adaptive immune responses are involved in all stages of atherosclerotic plaque development (1). Specifically, cytokines and cells belonging to the immunity promote endothelial dysfunction, a phenomenon that plays a crucial role at the beginning of the atherosclerotic plaque formation. Then, immunity seems to be involved in plaque grown and in its complications (1, 2). The term endothelial dysfunction indicates a particular vascular pathophysiological condition in which endothelial cells (ECs), as consequence of several stimuli, shift toward a pro atherothrombotic phenotype (3). Unperturbed ECs exert several “protective” functions since they modulate vascular tone, cellular adhesion, smooth muscle cell proliferation, and vessel wall inflammation (4). Importantly, healthy ECs have an active role in preventing thrombotic events by secreting several antithrombotic substances, such as PAI-1 and TFPI (5). However, it has been demonstrated that many stimuli can affect these antithrombotic functions of ECs, by modifying their gene expression profile and shifting them to a pro-thrombotic phenotype (6–11). Similarly, alterations of endothelial healthy status, by inducing the loss of its protective function, promote atherosclerosis (3). As reported above, several interesting evidence have revealed that, in this complex pathophysiological scenario, an important role might be ascribed to the modulation of endothelial gene expression (12, 13).

In the last decade, it has been demonstrated that activation of the immune system within the plaque should be the major determinant of its instability. In fact, unstable coronary plaques are infiltrated by macrophages (14), the prototypical cells of innate immunity, as well as by a larger quote of T-lymphocytes compared to stable plaques (15). T-cells appear to have a pivotal role in atherosclerotic plaque evolution because they regulate macrophage activity, cell to cell interaction, and production and release of cytokines (16), which may finally influence plaque vulnerability (17, 18). Indeed, it has been demonstrated that, in the clinical setting of acute coronary syndromes (ACS), a local cytokine storm, mainly related to Th response, may occur (19). Moreover, it has been shown that patients presenting higher coronary levels of specific subset of Th-levels and of Th-derived cytokines showed a worse disease course (20). Based on the available data, it has been suggested that acute coronary events might be associated to the local release of cytokines and other soluble factors that may influence plaque vulnerability because of a direct effect on the cellular components of the lesion. In line with these pathophysiological speculations, some studies have evaluated changes in gene profile during ACS, looking at systemic circulation (21, 22), carotid plaque (23), or platelets (24). However, no data are currently available on the effects of the soluble mediators released in the coronary circulation during the ACS and the gene expression profile in ECs from the coronary district. Thus, aim of the present study was to evaluate whether serum, and soluble mediators contained in it (19), obtained from the coronary circulation of ACS patients might cause changes in gene expression profile in coronary ECs *in vitro*.

## Materials and Methods

### Patient Population

Sixteen patients, divided in two groups, have been enrolled in the study. Group I included patients with chronic stable angina (SA, *n* = 4) undergoing elective coronary angiography; group II included patients with ACS-non-ST segment elevation myocardial infarction (NSTEMI) (*n* = 8), undergoing urgent coronary angiography and eventually percutaneous coronary intervention (PCI) within 24 ± 4 h from ACS diagnosis to avoid confounding parameters, mainly related to the timing of blood collection. All patients in group I had a history of effort chest pain with a stress test positive for inducible myocardial ischemia. ACS was defined as indicated in the current guidelines (25) with chest pain at rest occurring <48 h from hospital admission with ECG changes suggesting myocardial ischemia, with or without increase in serum markers of myocardial damage (troponin was measured at admission in all patients as standard care). Within the diagnosis of ACS, normal or increased serum markers of myocardial necrosis defined unstable angina (UA) or NSTEMI, respectively. UA was diagnosed in two patients, while the remaining eight patients had NSTEMI. Patients with previous myocardial infarction were excluded as well as patients with ST segment elevation myocardial infarction (STEMI) were excluded from the study. ACS–STEMI condition is characterized by a thrombotic formation that completely occludes the vessel with no distal flow. Thus, blood collected from coronary sinus (CS) in this condition might not be from the culprit vessel. As per protocol design, in both groups, only patients with TIMI flow grade ≥2 were included, as well as patients with a single vessel disease and a culprit lesion located either in the left anterior descending coronary artery or in the circumflex coronary artery, both of which drain into the CS.

The protocol was approved by the local ethical committee, and all patients gave written informed consent to participate to the study.

### Sample Collection

After coronary angiography was performed, and before performing PCI, a 6 F multipurpose catheter was positioned into the CS. To avoid that the different positions of the catheter might affect the blood sample, we have checked with angiography its position in the CS (Figure S1 and S2 in Supplementary Material). Blood samples were simultaneously obtained from the CS and the ascending aorta (Ao) and immediately placed into empty pre-chilled Vacutainer^®^ tubes for serum separation. Blood samples were immediately centrifuged at 1,000 × *g* at 4°C for 20 min, and the sera were stored in aliquots at −80°C.

### Cell Culture

Human coronary artery endothelial cells (HCAECs), endothelial basal medium-2 (EBM-2), EGM-2-MV SingleQuots, fetal bovine serum (FBS), and phosphate-buffered saline (PBS) were purchased from Lonza (Viviere, Belgium). HCAECs were maintained in EBM-2 supplemented with EGM-2-MV SingleQuots (containing vascular endothelial growth factor, basic fibroblast growth factor, insulin-like growth factor-I, epidermal growth factor, ascorbic acid, and gentamicin), and 10% FBS. All cells were cultured at 37°C in an incubator with a humidified atmosphere and 5% CO_2_. Cells were split at the ratio of 1:3 every passage. Cells from three to six passages were used in this study. For experiments, HCAECs were grown to confluence, washed with phosphate buffer (PBS), and kept in serum-free medium for 24 h to synchronize the cells, bringing them all to one phase of cell cycle. Afterward, cells were stimulated with medium containing 20% serum collected from Ao or CS of ACS or SA patients for 12 h and then total RNA was isolated following standard procedure and stored at −80°C for further analysis. All experiments have been performed in triplicates.

### Microarray Gene Expression Analysis

Agilent microarray analyses were done to evaluate the gene expression profile in HCAECs exposed to serum obtained from CS and Ao of patients with ACS and SA. These experiments were performed using a one color labeling microarray system. The quantity of each of the total RNA samples and determination of the A260/280 nm ratio was determined by spectrophotometry and the size distribution was assessed using an Agilent Bioanalyzer. Eight hundred nanogram of total RNA were converted into labeled cRNA with nucleotides coupled to a fluorescent dye (either Cy3) using the Quick Amp Kit (Agilent Technologies, Palo Alto, CA, USA) following the manufacturer’s protocol. The A260/280 nm ratio and yield of each of the cRNAs were determined using a Thermofisher Nanodrop.

Eight hundred twenty-five nanogram of cRNA-labeled from SC or Ao stimulated cells were hybridized to Agilent Human Whole Genome 4 × 44 k Microarrays. The hybridized array was washed and scanned, and data were extracted from the scanned image using Feature Extraction version 10.2 (Agilent Technologies). The raw data and associated sample information were loaded and processed by Gene Spring^®^ 11.5X (Agilent Technologies). For identification of genes significantly altered in patients with UA, total detected entities were filtered by signal intensity value (upper cut-off 100th and lower cut-off 20th percentile) and flag to remove very low signal entities. Data were analyzed using Student’s *t*-test (*p* < 0.05) with a Bonferroni multiple test correction to minimize selection of false positives. Of the significantly differentially expressed RNA, only those with greater than twofold increase or twofold decrease as compared to controls were used for further analysis. Subsequently, hierarchical clustering (condition tree) was applied to the data files. In this way, the relationships between the different groups are shown. The condition tree was displayed as a heat map, based on expression levels of the probe sets. Functional and network analyses of statistically significant gene expression changes were performed using Ingenuity Pathways Analysis (IPA) 8.0 (Ingenuity^®^ Systems, http://www.ingenuity.com). Analysis considered all genes from the data set that met the twofold (*p*-value < 0.05) change cut-off and that were associated with biological functions in the Ingenuity Pathways Knowledge Base. The significance of the association between the data set and the canonical pathway was measured in two ways: (1) ratio of the number of genes from the dataset that map to the pathway divided by the total number of genes that map to the canonical pathway is displayed and (2) Fisher’s exact test was used to calculate a *p*-value determining the probability that the association between the genes in the dataset and the canonical pathway is explained by chance alone.

### Real-time PCR

Real-time PCR has been performed on selected target genes. Following the manufacturer’s instructions, total RNA was extracted from HCAECs exposed to serum obtained from CS of patients with unstable and SA by using the RNAeasy Micro Kit (QIAGEN). The RNA obtained was treated with DNAase I to eliminate possible genomic DNA contamination. cDNA was synthesized with High-Capacity cDNA reverse transcription kits (Applied Biosystems). Total RNA (10 µL) was reversely transcribed in a total volume of 20 µL, containing 25× dNTP Mix (100 mM) 0.8 µL, 10× RT random Primers 2 µL, 10 × RT buffer 2 µL, MultiScribe reverse transcriptase 1 µL, RNase inhibitor 1 µL, DEPC free H_2_O add up to 3.2 μL. The reaction was run at 25°C for 10 min, 37°C for 120 min; 85°C for 5 min. The cDNA was stored at −20°C.

IL-17A, IL8, IL10, phospholipase C-beta 4 (PLCB4), PLA2 IIa, and PGE2genes were amplified by real-time PCR from mRNA using appropriate primers to detect their expression in HCAECs exposed to sera obtained from CS of patients with ACS or SA, respectively (Table S1 in Supplementary Material).

Real-time PCR and data analysis were performed using 96-microwell plates and a Biorad detector (Biorad). Two hundred nanogram of purified DNA, 5 µL of 5× Hot TaqEvaGreen (Microtech), and 250 nM of primers were added to each microwell to reach a total volume of 20 µL per well, DNase-RNase-free distilled water (Sigma) was added. The reaction was run at 95°C for 15 min, followed by 40 cycles at 95°C for 15 s, 60–65°C for 20 s and 72°C for 20 s. Three housekeeping genes were used as a control following the latest MIQE guidelines (26). The specificity of the amplification products was controlled using a melting curve analysis. The copy number of IL-17A, IL-8, IL-10, PLCB4, PLA2 IIa, prostaglandin E2 (PGE2), and housekeeping transcripts in samples was calculated with the Bio-Rad CFX Manager software according to corresponding standard curves. All samples were measured in triplicate. Data are presented as mean ± SEM. Differences between groups were determined by a one-way ANOVA followed by a Student’s *t*-test with Bonferroni’s correction. A *p*-value < 0.05 was considered statistically significant.

## Results

### Patient Population

Clinical characteristics of the patients enrolled are reported in Table 1.

**Table 1:** **Demographic characteristics of study population**.

	Group I stable angina *n* = 4	Group II (acute coronary syndromes–non- ST segment elevation myocardial infarction) *n* = 8
Age (years)	60 ± 11	63 ± 12
Male sex (%)	2 (50)	4 (50)
# of diseased vessels per patient	1	1
Left anterior descending artery	2 (50)	4 (50)
Circumflex artery	2 (50)	4 (50)
Major risk factors (%)		
Hypercholesterolemia	4 (100)	7 (87.5)
Hypertension	4 (100)	8 (100)
Diabetes	2 (50)	4 (50)
Smoking	3 (75)	6 (75)
Medications (at time of angiography)		
β-blockers	2 (50)	6 (75)
ACE-Inhibitors	3 (75)	6 (75)
Angiotensin II receptor blockers	1 (25)	2 (25)
Statins	4 (100)	10 (76,9)
Thienopyridines	1 (25)	2 (25)
Aspirin	1 (25)	5 (62,5)
Nitrates	3 (75)	2 (25)

### Microarray Gene Profile

The expression profile of ACS-related genes was determined by comparing gene expression in HCAECs exposed to serum collected from Ao and CS of patients with SA. To exclude that serum exerted direct toxic effects on HCAECs, trypan blue exclusion assays were performed at conclusion of the incubation and demonstrated >95% viability with no differences between the different experimental groups (data not shown).

**Figure 1:**
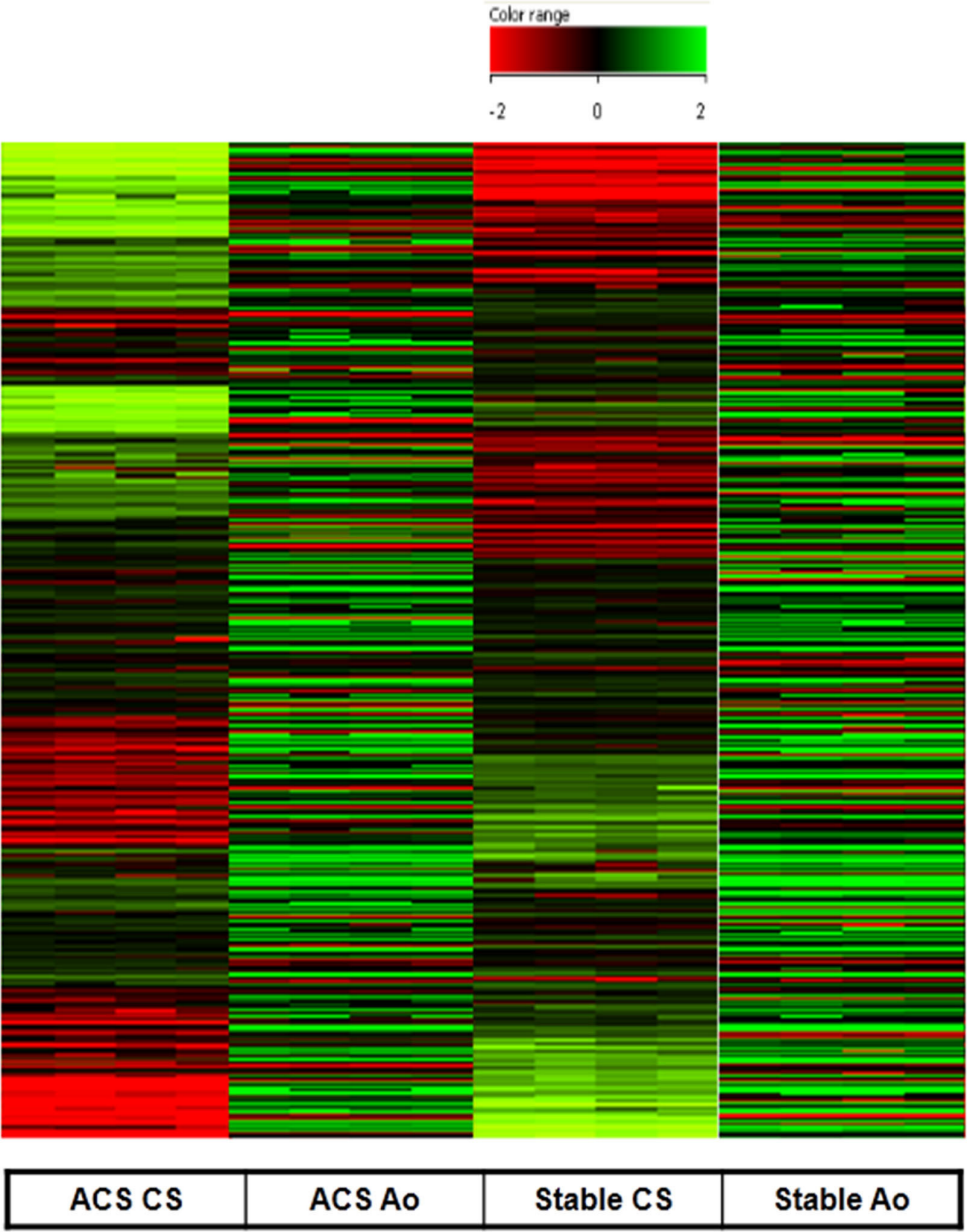
**Gene expression measured in human coronary artery endothelial cells stimulated with serum from aorta and coronary sinus of patients with acute coronary syndromes (ACS) and stable angina (SA)**. Upregulated (684) genes are represented in green and downregulated (283) are represented in red (difference ≥2-fold, or ≤-2-fold were measured). All microarray data are registered in GEO platform with the following code GSE54977 (different gene expression profile in ACS patients compared to SA patients), and are available at the link: http://www.ncbi.nlm.nih.gov/geo/query/acc.cgi?acc=GSE54977.

As expected, serum obtained from the Ao of ACS patients did not determine any significant change in gene expression profile as compared to serum obtained from the Ao of SA patients. In contrast, serum collected from the CS of patients with ACS caused a significant change in gene expression profile with respect to CS serum samples from patients with SA (Figure 1). Specifically, only genes with twofold increase or decrease were considered significant (Tables 2 and 3). Among the differentially expressed genes, 684 were upregulated and 283 downregulated (Figure 1). The genes with a statistically significant variation underwent to functional and network analyses using IPA 8.0.

**Table 2:** **Upregulated genes in HCAECs stimulated with serum from Cs of acute coronary syndromes patients**.

Gene symbol	Gene name	*p*-Value	Fold change
SELE	Selectin E	3.01E−10	19.661016
CXCL1^a^	Chemokine (C–X–C motif) ligand 1 (melanoma growth stimulating activity, alpha)	1.05E−19	8.485691
CXCL2	Chemokine (C–X–C motif) ligand 2	3.95E−25	6.6117725
CCL2^a^	Chemokine (C–C motif) ligand 2	2.75E−20	6.3248153
PTGS2^a^	Prostaglandin-endoperoxide synthase 2 (prostaglandin G/H synthase and cyclooxygenase)	3.53E−28	5.8133426
CD86	CD86 molecule	1.34E−06	4.9321218
RELB	v-Rel reticuloendotheliosis viral oncogene homolog B	1.63E−20	4.7633815
FOSB	FBJ murine osteosarcoma viral oncogene homolog B	4.75E−15	4.325817
NFKBIA	Nuclear factor of kappa light polypeptide gene enhancer in B-cells inhibitor, alpha	3.81E−17	4.0123706
JUNB^a^	Jun-B protoncogene	1.07−08	3.95
ID1	Inhibitor of DNA binding 1, dominant negative	1.14E−15	3.679152
NFKBIZ	Nuclear factor of kappa light polypeptide	5.64E−23	3.3618975
FOS	FBJ murine osteosarcoma viral oncogene homolog	1.12E−16	3.2505574
FGF18	Fibroblast growth factor 18	5.17E−12	3.2300966
IL7R	Interleukin 7 receptor	8.03E−07	3.1095386
ADAMTS1	ADAM metallopeptidase with thrombospondin type 1 motif, 1	4.85E−20	3.1251428
CD83	CD83 molecule	2.72E−12	2.8649588
IL8^a^	Interleukin 8	1.22E−17	2.8357644
IL11	Interleukin 11	4.98E−11	2.8224506
CXCL3	Chemokine (C–X–C motif) ligand 3	2.48E−13	2.7130814
ICAM1	Intercellular adhesion molecule 1	1.46E−20	2.6648185
FOSL2	FOS-like antigen 2	7.37E−21	2.5525434
TRAF1	TNF receptor-associated factor 1	1.02E−09	2.4518566
NFKBIE	Nuclear factor of kappa light polypeptide	4.15E−24	2.3899362
VEGFA	Vascular endothelial growth factor A	1.12E−14	2.3613784
HBEGF	Heparin-binding EGF-like growth factor	1.68E−18	2.3418474
RASSF8^a^	Ras association domain family	6.72E−06	2.34
MAP3K8	Mitogen-activated protein kinase kinase kinase 8	2.69E−16	2.270313
IL3RA	Interleukin 3 receptor, alpha (low affinity)	2.29E−10	2.2516959
VASP	Vasodilator-stimulated phosphoprotein	7.61E−08	2.2024417
IL6	Interleukin 6	1.44E−08	2.20
GPR68	G protein-coupled receptor 68	0.0041	2.16986
BMPR1B	Bone morphogenetic protein receptor, type IB	5.94E−05	2.12
FBLN1	Fibulin 1	3.95E−07	2.1075332
RASL10A^a^	RAS-like, family 10, member A	2.60E−13	2.10
PF4	Platelet factor 4	0.0043	2.0621264

*^a^Gene involved in IL-17 signaling*.

**Table 3:** **Downregulated genes in HCAECs stimulated with serum from Cs of acute coronary syndromes patients**.

Gene symbol	Gene name	*p*-Value	Fold change
CXCR4	Chemokine (C–X–C Motif) receptor 4	3.44E−26	−4.4
IL 10	Interleukin 10	2.63E−0.7	−4.15
CCNG2	Cyclin G2	4.98E−12	−3.5
CDKN1B	Cyclin-dependent kinase inhibitor 1B	6.43E−24	−2.95
CFH	Complement factor H	2.68E−13	−2.59
IL 33	Interleukin 33	2.39E−12	−2.43
NAMPT	Nicotinamide phosphoribosyltransferase	6.88E−07	−2.15
TBET	T-cell-specific T-Box transcription factor T-Bet	1.01E−11	−2.14
STAT1	Signaling transducer and activator of transcription-1	1.36E−11	−2.12
RAB5A	RAB5A, member RAS oncogene family	5.37E−20	−2.09
CCNG1	Cyclin G1	2.43E−11	−2.04
SENP7	SUMO1/sentrin specific peptidase 7	1.30E−07	−2.04
IL 5	Interleukin 5	3.74E−08	−2.03
VLDLR	Very low density lipoprotein receptor	1.18E−12	−2.01
TNFSF10	Tumor necrosis factor (ligand) superfamily, member 10	4.68E−15	−2.00

The E-selectin (SELE) gene was the most upregulated one, followed by Chemokine (C–C motif) ligand 2 (CCL2), Chemokine (C–X–C motif) ligand 1 (CXCL1), and prostaglandin G/H synthase and cyclooxygenase (PTGS2) genes. Analysis of the upregulated genes in HCAECs stimulated with CS serum from ACS patients were associated with the following pathways: TNF receptor-2 signaling, CD40 signaling, NFKB signaling, glucocorticoid receptor signaling, B cell activating factor signaling, MIF regulation of innate immunity, dendritic cell maturation, factors promoting cardiogenesis in vertebrate, TGF-beta signaling, PKCθ signaling in T lymphocytes, April-mediated signaling, cardiomyocyte differenzation *via* BMP receptor, granzyme A signaling, IL8 signaling, HMGB1 signaling, hypoxia signaling in the Cardiovascular System, TREM1 signaling, TWEAK signaling, RAR activation, PI3K signaling in B lymphocytes, lymphotoxin in B receptor signaling, BMP signaling, MIF-mediated glucocorticoid regulation, TNRF1 signaling, 4-IBB signaling in T Lymphocytes, ATM signaling, and CD27 signaling in Lymphocytes (Table 4). On the contrary, in the same experimental set-up the downregulated genes were associated with the following pathways: OX40 signaling, PI3K/AKT signaling, G α 12/13 signaling, Toll-like receptor signaling, regulation of IL2 expression in activated and anergic T lymphocytes, TIGHT junction signaling, CD28 signaling in T-helper cells, B-cells signaling, erytropoietin signaling, ILK signaling, IL10 signaling, FGF signaling (Table 5).

**Table 4:** **Upregulated pathways in HCAECs stimulated with serum from Cs of acute coronary syndromes patients**.

PATWAYS	*p*-Value
TNRF2 signaling	9.32E−6
CD40 signaling	9.07E−4
NFkB signaling	8.14E−3
IL17 signaling	7.29E−4
Glucocorticoid receptor signaling	6.13E−4
B cell activating factor signaling	5.9E−3
MIF regulation of innate immunity	5.38E−3
Dendritic cell maturation	5.11E−3
Factors promoting cardiogenesis in vertebrates	5.11E−3
TGFB signaling	5.00E−4
PKCθ signaling in T lymphocytes	4.92E−2
April mediated signaling	4.9E−3
MSP-RON signaling pathway	4.86E−2
Communication between innate and adaptive immune cells	4.81E−2
Protein kinase A signaling	4.52E−2
Cardiomyocytes differenziation *via* BMP receptor	3.99E−3
Granzyme A signaling	3.76E−2
IL 8 signaling	3.74E−2
HMGB1 signaling	3.39E−4
Hyproxia signaling in the cardiovascular system	3.08E−2
TREM1 signaling	2.96E−3
TWEAK signaling	2.92E−3
RAR activation	2.91E−3
PI3K signaling in B lymphocytes	2.88E−2
Lymphotoxin B receptor signaling	2.01E−2
BMP signaling	1.98E−3
MIF-mediated glucocorticoid regulation	1.85E−2
TNRF1 signaling	1.59E−3
4-IBB signaling in T-lymphocytes	1.54E−2
ATM signaling	1.47E−2
CD 27 signaling in lymphocytes	1.38E−2

**Table 5:** **Downregulated pathways in HCAECs stimulated with serum from Cs of acute coronary syndromes patients**.

Pathways	*p*-Value
OX40 signaling	4.75E−2
PI3K/AKT signaling	4.39E−2
G α 12/13 signaling	4.39E−2
Toll-like receptor signaling	4.39E−2
Regulation of IL2 expression in activated and anergic T-Lymphocytes	4.22E−2
TIGHT junction signaling	4.11E−2
CD28 signaling in T-helper cells	3.99E−2
B-cells signaling	3.19E−2
Erythropoietin signaling	2.89E−2
ILK signaling	2.75E−2
IL 10 signaling	2.51E−2
FGF signaling	1.35E−2

Analysis of the downregulated genes revealed that the most modulated ones were the Chemokine (C–X–C motif) receptor 4 (CXCR4) and the IL-10.

**Figure 2:**
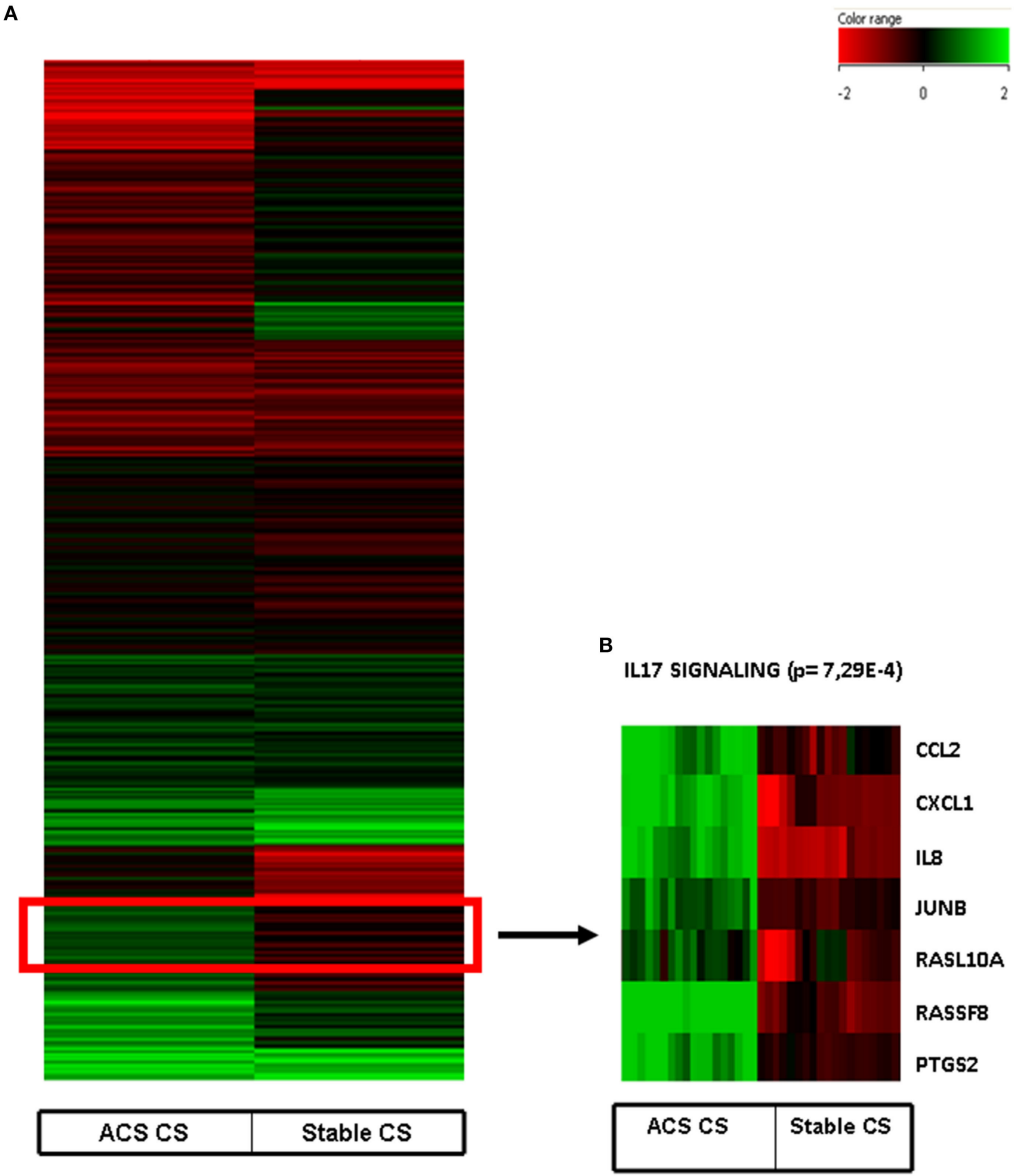
**(A)** Heat map of human coronary artery endothelial cells stimulated with serum obtained from coronary sinus of patients with acute coronary syndromes and normalized with human coronary endothelial cells incubated with serum obtained from aorta of the same patients. **(B)** Heatmap of IL-17 pathway (*p* = 7.29E−4) obtained after Ingenuity Pathways Analysis.

Interestingly, a subanalysis of the upregulated genes revealed that a cluster of them was associated with the pathways of IL-17 Signaling (Figure 2): chemokine (C–C motif) ligand 2 (CCL2), chemokine (C–X–C motif) ligand 1 (CXCL1), interleukine 8 (IL8), Janus kinase 1, v-Ki-ras2 Kirsten rat sarcoma viral oncogene homolog, muscle RAS oncogene homolog, prostaglandin-endoperoxide synthase 2, prostaglandin G/H synthase and cyclooxygenase (PTGS2) as shown in Table 3 and Figure 2.

### Real-time PCR

Since we have previously demonstrated that Th-17 and its derived interleukin, IL-17, might be involved in the ACS pathophysiology (20), we pointed out our attention on this specific pathway.

**Figure 3:**
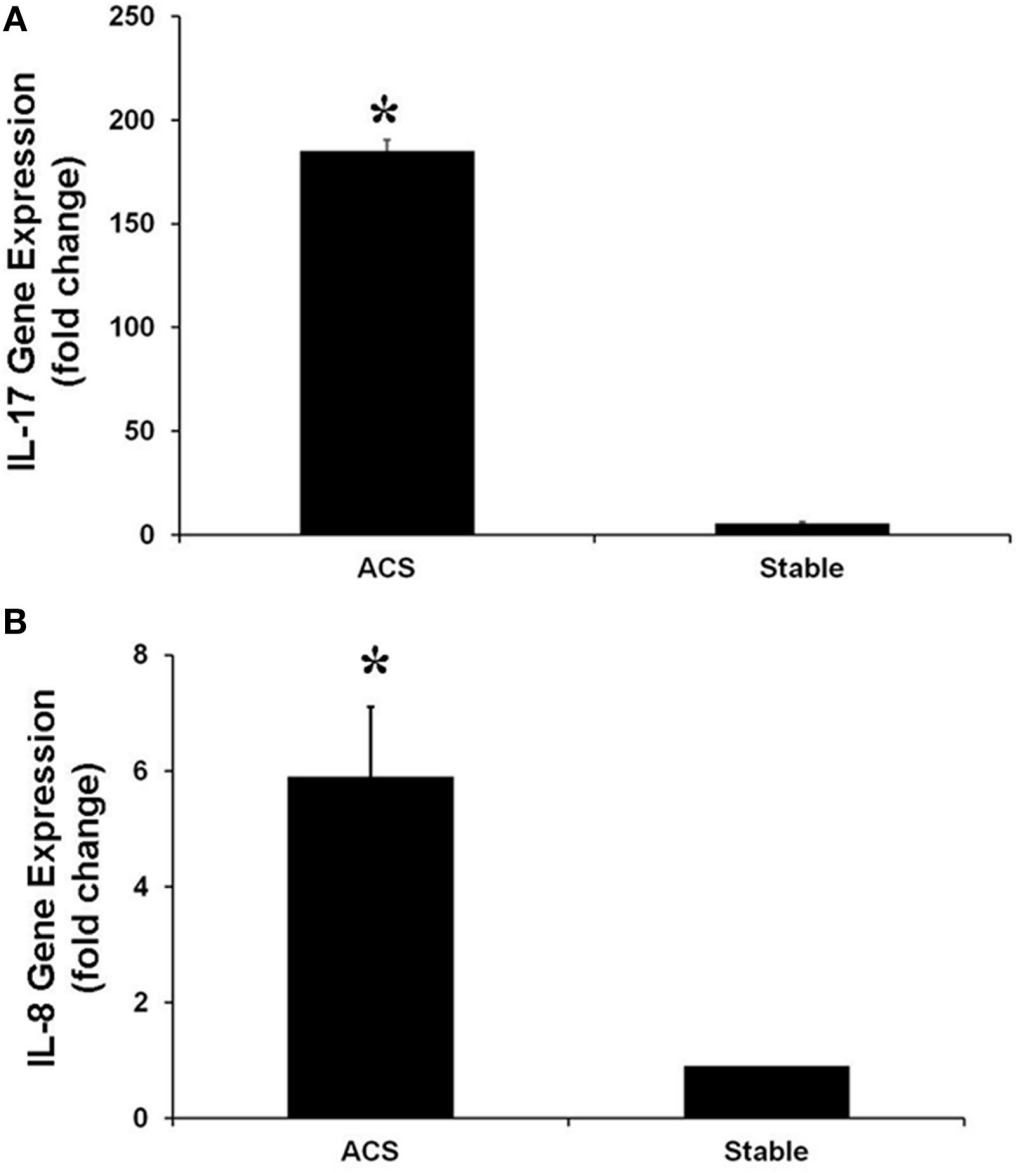
**Gene expression of IL-17A (A) and IL-8 (B) genes in Human Coronary Endothelial Cells stimulated for 12 h with serum obtained from coronary sinus (CS) of patients with acute coronary syndromes, or from CS of patients with stable angina determined by real-time PCR analysis**. Data are expressed as the mean ± SEM of three independent experiments (*p < 0.05).

**Figure 4:**
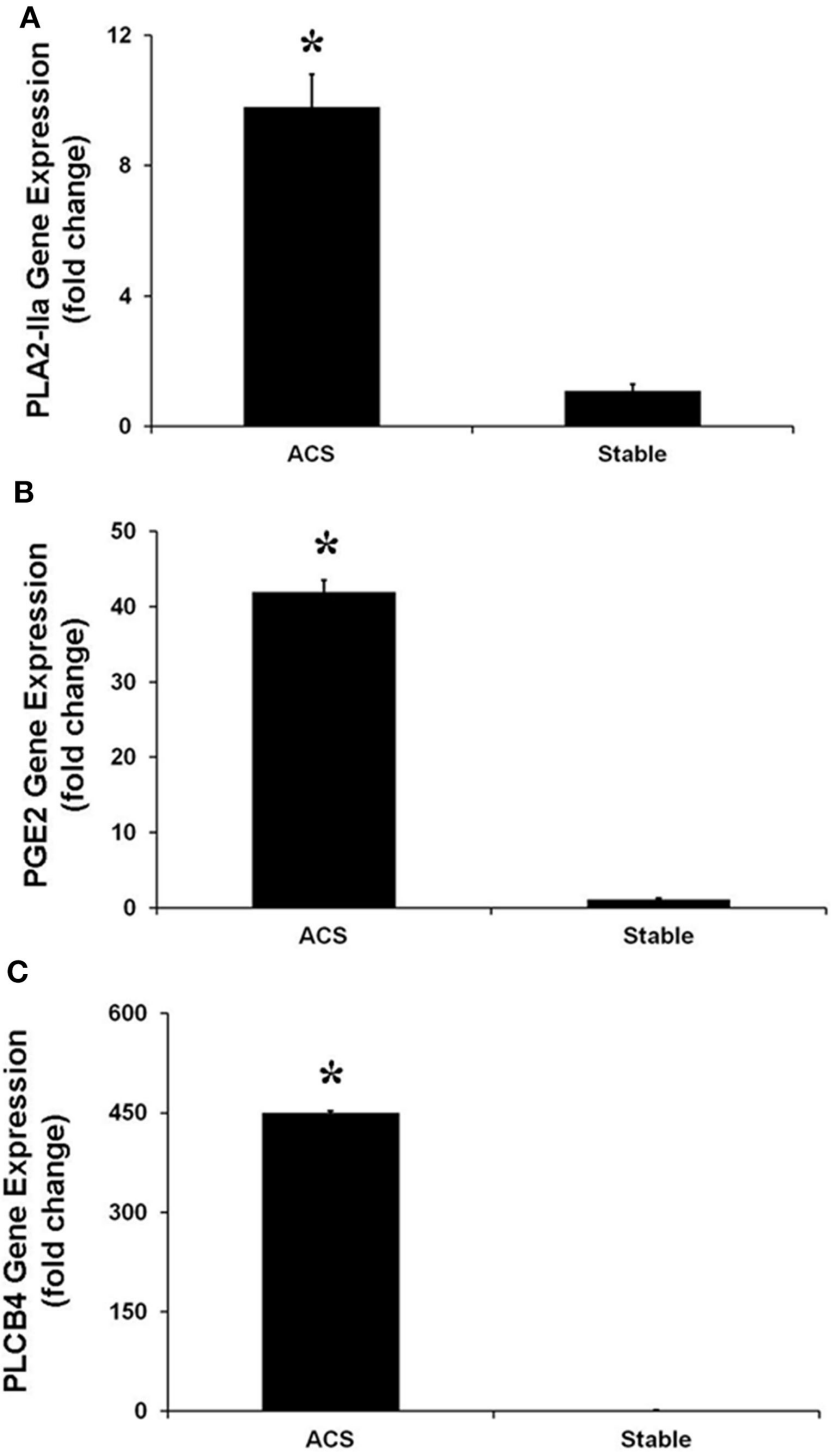
**Gene expression of PLA-IIA (A), prostaglandin E2 (B), and phospholipase C-beta 4 (C), in Human Coronary Artery Endothelial cells stimulated for 12 h with serum obtained from coronary sinus (CS) of patients with acute coronary syndromes, or from CS of patients with stable angina determined by real-time PCR analysis**. Data are expressed as the mean ± SEM of 3 independent experiments (*p < 0.05).

Real-time PCR experiments were performed to evaluate the expression of several genes belonging to the IL-17 pathway. Specifically, the levels of IL-17A, which is mainly produced by Th17 cells, as well as the levels of phospholipase A2 (PLA2 IIa), PLCB4 and their product PGE2 levels, involved in the activation of Th17 cells, were measured. In HCAECs stimulated with serum from the CS of ACS patients a significant increase of RNA levels for IL-17A, PLA2 IIa, PLCB4, and PGE2 was observed as compared to cells stimulated with serum from SA patients (Figures 3 and 4). Moreover, the levels of IL-8 were increased (Figure 3).

**Figure 5:**
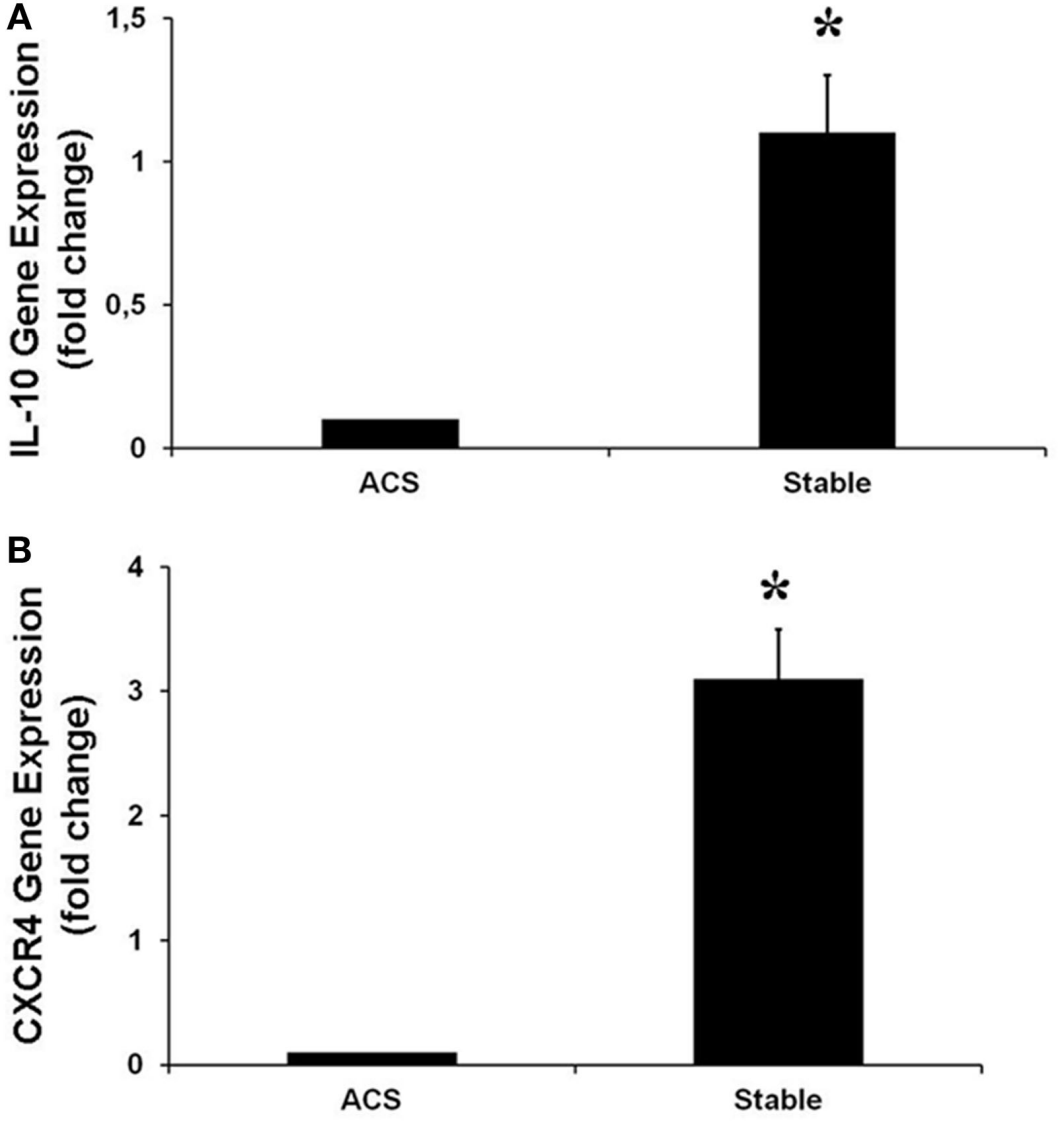
**Gene expression of IL-10 (A), CXCR4 (B), in human coronary artery endothelial cells stimulated for 12 h with serum obtained from coronary sinus (CS) of patients with acute coronary syndromes, or from CS of patients with stable angina determined by real-time PCR analysis**. Data are expressed as the mean ± SEM of three independent experiments (*p < 0.05).

Real-time PCR was performed to evaluate the levels of expression of CXCR4 and IL-10, the most downregulated genes. Interestingly, in HCAECs stimulated with serum from CS of ACS patients, levels of expression for these genes were significantly lower as compared with the expression measurable in cells stimulated with serum from CS of SA patients (Figure 5).

## Discussion

In the present study, we show that sera collected from the CS of ACS patients, which resemble coronary circulation, induce a strong modulation at gene levels in human coronary endothelial cells, which results in upregulation of 684 genes and downregulation of 283 genes, as compared to cells stimulated with sera from SA patients. On the contrary, sera collected from the Ao of these patients, which resemble the systemic circulation, do not determine any significant change in gene expression profiles in human ECs compared to patients with SA. These results suggest that, during an acute event, in the coronary circulation there are “local” factors able to affect gene expression profile of coronary ECs. These changes of endothelial gene profile seem associated with modulation of cellular pathways that might facilitate the progression of coronary atherosclerosis and lead to acute complications such as acute coronary events.

We have observed upregulation of many genes that are known to be involved in the early steps and in the progression of atherosclerosis. Specifically, SELE, the most upregulated gene, is a glycoprotein actively involved in the first steps of atherosclerosis since it sustains the leukocytes adhesion to endothelial wall and their passage to the subendothelial space (27, 28). Moreover, we observed the upregulation of genes that codify for several chemokines such as PTGS2, CCL2, and CXCL1 and 2, that, in response to pro-inflammatory cytokines, promotes the recruitment of other effector cells, such as monocytes, granulocytes, and T cells-effector, actively involved in the progression of atherosclerosis (29–31). Furthermore, other upregulated genes were those of the pro-inflammatory interleukins IL3RA, IL7R, IL8, IL11, usually secreted by immune system cells, and of the NF-κB family (NF-κBIA, NF-κBIZ, NF-κBIE), nuclear transcription factors known to be involved in the development of atherosclerosis (32, 33). Again, many other upregulated gene in coronary ECs, such as PTGS2, fibroblast growth factor 18 (FGF18), vascular endothelial growth factor A (VEGF-A), intercellular adhesion molecule 1 (ICAM1), or platelet factor 4 (PF4), are selectively involved in the inflammatory pathways as mechanisms responsible of atherosclerosis (34–37).

We have recently demonstrated that a cytokine storm occurs in the coronary circulation of patients with ACS, reflecting mainly a Th1 response (19). Moreover, we have shown that ACS patients with higher levels of Th17, a separate CD4^+^ T cell subset distinct from Th1, Th2, had a worse clinical outcome, suggesting that these T cells might be actively involved in ACS pathophysiology (20). In line with these previous observations, in the present report, we indicate that, in HCAECs stimulated with serum from CS of ACS patients, among several upregulated genes, some of them were associated with the pathways of IL-17. Here, we demonstrate that levels of gene expression for IL-17A that is mainly produced by Th17 cells are elevated in HCAECs stimulated with serum from coronary circulation of ACS patients. The IL-17A receptor is ubiquitously expressed and the cytokine has several complex activities: it promotes the production of TNF- β, IL-1 β, and MCP-1 as well as of adhesion molecules like ICAM1 (38, 39). Several studies already showed that the IL-23/IL-17 axis acts as a bridge between the innate and the adaptive immune system (40). Specifically, IL-17 is involved in the response against extracellular bacteria and fungi regulating neutrophil recruitment and activation, and it is essential in infectious and autoimmune diseases pathogenesis (41). In the cardiovascular system, IL-17A, by acting on several cell populations actively involved in pathophysiology of athero-thrombosis such as macrophages and monocytes, or normally represented in the vascular wall, such as endothelial and smooth muscle cells, is able to induce a pro-inflammmatory status, thrombosis and to destabilize plaque (41). Moreover, elevated levels of IL-17A expression have been detected in ruptured or lipid-rich plaques (42).

Besides evaluating gene expression for IL-17A, that is mainly produced by Th17 cells, we have measured the expression of some cytokines involved in Th17 cells activation such as PLA2 IIa, PLCB4 and their product PGE2 (43–45). In HCAECs stimulated with serum from CS of ACS patients, we observed the significant increase of gene expression for PLA2IIa, PLCB4 and PGE2 as compared to cells stimulated with serum from CS of AS patients. Taken together, and in line with previous reports about the role of Th-17/IL-17 in ACS pathophysiology (19, 46), these results suggest a potential relationship between changing of HCAECs gene profile in response to inflammatory stimuli and future coronary events. Specifically, dysfunctional ECs might themselves produce IL-17 and, at the same time, significantly enhance Th-17 response in the coronary circulation, finally creating a pro-atherothrombotic milieu.

Another interesting finding was that in HCAECs stimulated with CS sera from ACS patients, a significant downregulation of IL-10 gene was observed. Interleukin 10 is a kind of an anti-inflammatory cytokine which can reduce the formation of atherosclerosis and maintain the stability of atheromatous plaques, which play an important role in inhibiting the occurrence of ACS (46–48). Thus, these protective, anti-inflammatory properties of IL-10 seem to be significantly reduced in ECs as consequence of soluble mediators contained in the sera from the coronary circulation of ACS patients.

Finally, we have found that, in HCAECs stimulated with serum form ACS patients, the levels of CXCR4 expression were significantly lower than those measurable in cells stimulated with serum from SA patients. CXCR4 is a receptor for the chemokine CXCL12 (also known as stromal cell derived factor-1, SDF-1), widely and constitutively expressed by hematopoietic and ECs (49). The SDF-1/CXCR-4 pathway is a prerequisite of embryonic vasculogenesis, and induction of SDF-1 expression seems to have a major role in initiating revascularization of the ischemic injured tissues (50, 51). Thus, it might be speculated that soluble mediators released in the coronary circulation during an ACS, by reducing CXCR4 expression in ECs, might affect also the mechanisms involved in myocardial revascularization and, specifically, the healing of the injured vessel.

### Potential Limitations of the Present Study

Some potential limitations should be taken in account in evaluating the results of the present study. First, the number of patients might be considered too small, however, this is a research study with pathophysiological aims. Thus, for such experimental studies, we do need of a small, but statistically significant number of observations. Another potential limitation is that, in addition to taking serum, it would be useful to perform also flow cytometric analysis of T-cell immunity in order to correlate the results with immune function. Indeed, patients of the present study have been already studied in their immunological profile in a previous report from our research group (19). Specifically, the present study has been performed by selecting those patients with highest trans-coronary levels of Th17/IL-17.

## Conclusion

**Figure 6:**
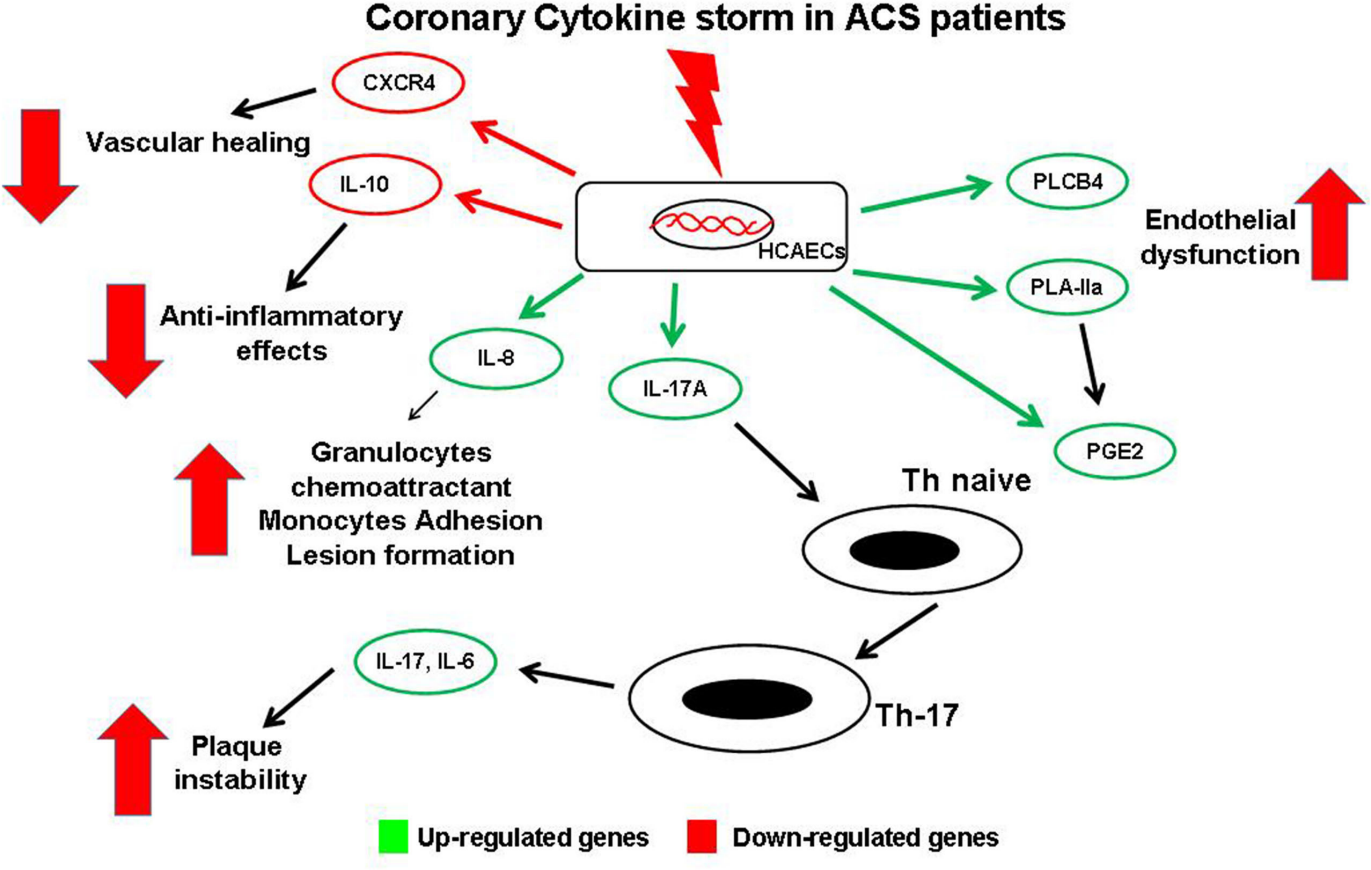
**Schematic view of gene expression profile in endothelial cells (ECs) and possible interaction with T-cells**. Soluble mediators released in coronary circulation of acute coronary syndromes patient modulate changes of gene expression profile in coronary ECs. Several genes are upregulated, causing a local increase of of some cytokines. IL-17 may induce differentiation of naive T-cells into Th-17cells, thus promoting plaque destabilization. Similarly, increased expression of phospholipase C-beta 4, PLA-IIA, and prostaglandin E2 enhance coronary endothelial dysfunction and local Th-17 recruitment. Moreover, high levels of IL-8 induce atherosclerotic lesion formation. Conversely, other genes are downregulated with decreased levels of the protective chemical mediators such as the anti-atherosclerotic cytokine IL-10, and the CXC-R, involved in vessel healing.

Results of the present study, with all potential limitations derived from an *in vitro* study, highlight how, during an acute event, the chemical mediators released in the coronary circulation might be able to perturb coronary ECs modifying their gene profile. These modified ECs, through downregulation of protective gene and, mainly, through upregulation of gene able to modulate the Th-17/IL-17 pathway, might play a key role in progression of coronary atherosclerosis and in developing future acute events (Figure 6). Although further studies are required to confirm these findings in clinical settings, this study contributes to shed a brighter light on the complex relationship between ECs and immunity in the coronary district in the complex scenario of ACS pathophysiology.

## Author Contributions

GCimmino and PCirillo, wrote the manuscript and contributed to data interpretation. LC and GCiccarelli performed cell culture and cell experiments. PCalabrò, PCirillo, and PG screened the patients, performed coronary angiography and collected blood samples. FF, AR, and RP performed experiments of gene expression. PG, FR, PCirillo, and LB contributed to data interpretation, final draft, and gave the final acceptance.

## Conflict of Interest Statement

The authors declare that the research was conducted in the absence of any commercial or financial relationships that could be construed as a potential conflict of interest.

## Supplementary Material

Figures S1 and S2 **Positioning of the 6 F multipurpose catheter into the coronary sinus**.

Table S1 **Primers sequence**.
