# Supplementary figures and images for: Upregulation of TH/IL-17 Pathway-Related Genes in Human Coronary Endothelial Cells Stimulated with Serum of Patients with Acute Coronary Syndromes
Source: Front Cardiovasc Med. 2017 Feb 7;4:1. doi: 10.3389/fcvm.2017.00001 (PMC5293806; doi:10.3389/fcvm.2017.00001)

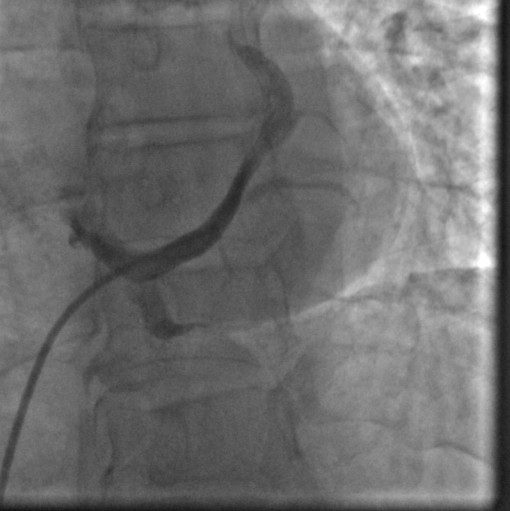

Supplement: Supplementary file 1 [file image_1.jpeg]

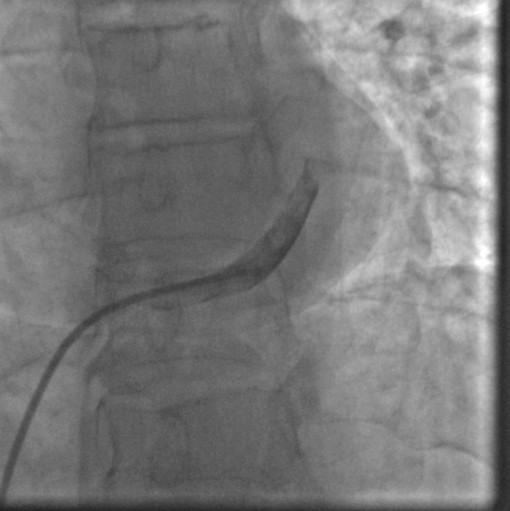

Supplement: Figures S1 and S2 — Positioning of the 6 F multipurpose catheter into the coronary sinus. [file image_2.jpeg]
