# Supplementary material for: Upregulation of TH/IL-17 Pathway-Related Genes in Human Coronary Endothelial Cells Stimulated with Serum of Patients with Acute Coronary Syndromes
Source: Front Cardiovasc Med. 2017 Feb 7;4:1. doi: 10.3389/fcvm.2017.00001 (PMC5293806; doi:10.3389/fcvm.2017.00001)
Supplement: Table S1 — Primers sequence. [file table_1.pdf]

Gene	Primers
IL17A	FOR: GGAATCTCCACCGCAATGA<br>REV: AGAGCTCTTAGGCCACATGGT
PGE2	FOR: GCATGTGTCATGACCTGGAC<br>REV: CCGGGAGAAATGATCAAAGA
PLA2-IIA	FOR: AAGGAAGCCGCACTCAGTTA<br>REV: TTGCACAGGTGATTCTGCTC
PLA2-IVA	FOR: CCCGACTTATTTGGAAGCAA<br>REV: GGAGCCTCTGCTTTGTGAAC
PLCB4	FOR: CGTGGCTTTCCAAGAAGAAG<br>REV: GCTTCCGATCTGCTGAAAAC
IL8	FOR: AGACATACTCCAAACCTTT<br>REV: GCTCTCTTCCATCAGAAA
IL10	FOR: CTCTGCTGAAGGCATCTCGG<br>REV: GTCCTCCTGACTGGGGTGAG

Supplementary Table S1: Primers sequence
